# ANATOMIC STUDY OF THE NERVOUS COMMUNICATION BETWEEN THE MEDIAN AND MUSCULOUCUTANEOUS NERVE

**DOI:** 10.1590/1413-785220162404159372

**Published:** 2016

**Authors:** Edie Benedito Caetano, Luiz Ângelo Vieira, Cristina Schmitt Cavalheiro, Mauro Razuk, Marco Antonio Pires Almargo, Mauricio Ferreira Caetano

**Affiliations:** 1. Pontifícia Universidade Católica de São Paulo, Faculdade de Ciências Médicas e da Saúde, Department of Surgery. Sorocaba, SP, Brazil.; 2. Pontifícia Universidade Católica de São Paulo, Faculdade de Ciências Médicas e da Saúde, Campus Sorocaba, Sorocaba, SP, Brazil.

**Keywords:** Musculocutaneous nerve, Median nerve, Anastomosis, surgical/methods, Nerve transfer, Ulnar nerve

## Abstract

**Objective:**

The aim of this study was to analyze the incidence of nerve communication between the musculocutaneous and median nerve***.***

**Methods:**

Anatomical dissection of 40 limbs from 20 fetal cadavers was performed at the Laboratory of Anatomy, Faculdade de Ciências Médicas e da Saúde da Pontifícia Universidade Católica de São Paulo***.***

**Results:**

A communicating branch was found in 10 upper limbs. In nine limbs there was a musculocutaneous-median anastomosis (type I); and in one limb there was a median-musculocutaneous anastomosis (type II)***.***

**Conclusion:**

It is very important to know these anatomical variations, especially when considering clinical examination, diagnostic, prognostic and surgical treatment. ***Level of Evidence IV, Case Series.***

## INTRODUCTION

From the brachial plexus toward the hand, we can find anomalous nerve branches, which can form anastomoses in peculiar places that have clinical and functional relevance. The nerve communication (anatomical variations) between the median and ulnar nerve in the forearm (Martin Gruber anastomosis),^1^ between the thenar motor branch of the median nerve and the deep branch of the ulnar nerve in the palm of the hand (Cannieu and Riché anastomosis)^2^ have been described. Moreover, even between the sensory branches of both nerves in the hand palm (Berretini anastomosis)^3^ or superficial sensory communicating branch have been described with a certain frequency in the literature and has been the object of our studies.

These anatomical variations (nerve anastomoses) generate the transfer of fascicles between the nerves, causing change from the normal anatomical pattern of motor and sensory innervation. The literature shows that the incidence of nerve communication is variable when comparing different methods of investigation, namely by electromyography studies, selective anesthetic nerve block or by anatomical dissections. According to Sunderland,^4^ there are several factors that make it difficult to assess nerve function, the main factors are anatomic variations, or also the failure to evaluate the role of deceptive movements, because they allow imitate and cover up the loss of the original movements. If these factors are not valued, errors in diagnosis and evaluation of results will be inevitable.

The objective of this study is to demonstrate through anatomical dissections in the arms of fetuses, the presence of anastomoses (nerve communication) between the musculocutaneous and median nerves (MCN-MN).

The musculocutaneous nerve and the lateral root of the median nerve originate from the lateral cord of the brachial plexus. It is possible that in embryonic development some nerve fascicles that originally were part of the median nerve were transferred to the musculocutaneous nerve, and through these nerve communications in the arms, these fascicles are recovered by the median nerve.^5^ ^,^ ^6^

## MATERIALS AND METHODS

We dissected 40 arms of 20 fetuses' bodies (stillborn) from the Anatomy Laboratory of *Faculdade de Ciências Médicas e da Saúde da Pontifícia Universidade Católica de São Paulo, Campus Sorocaba.* Regarding gender, 13 corpses were male and seven female. Limbs deformed by trauma, malformations and scars were excluded.

A straight incision was made in the anterior compartment of the arm following the anterior midline, beginning in the supraclavicular region and ending in the cubital fossa. Two flaps including the skin and subcutaneous tissue were folded to the medial and lateral sides, respectively. The same was done in relation to the arm fascia, thereby exposing the whole musculature. Tenotomy of the major and minor pectoral muscles was performed, and the clavicle was removed for better exposure of the entire brachial plexus. Thus, it was possible to identify the medial and lateral fascicles.

Dissection was done from proximal to distal, following the median and musculocutaneous nerve, certifying the presence or absence of nerve communication. The arm length was measured from the acromion to the medial epicondyle of the humerus. The length of the anastomotic branch, as well as its location on the arm was also recorded. We used a surgical magnifying glass with an increase of 2.5 x 350 mm (Keeler brand) and a delicate surgical instrument box for dissection. At the end, all our dissections were photographically cataloged. This study was approved by the Ethics Committee of *Faculdade de Ciências Médicas e da Saúde da Pontifícia Universidade Católica de São Paulo, Campus Sorocaba* (PUCSP). 

## RESULTS

We dissected 40 arms from 20 fetuses and found 10 anastomoses between the median and musculocutaneous nerve, 9 anastomoses from the musculocutaneous nerve to the median nerve (90%) and one from the median nerve to the musculocutaneous nerve (10%). Therefore, in percentage figures we have: 25% of anastomosis between the median and musculocutaneous nerve and 75% of anastomosis absence.

**Figure 1:**
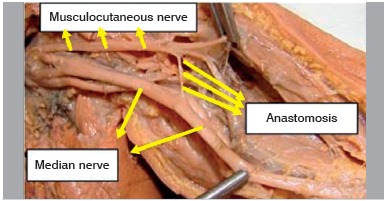
Specimen 8 - Left (presence of musculocutaneous-median anas tomotic branch).

Among the 10 anastomoses, we have: 6 anastomoses in the left arm (60%) and four anastomoses in the right arm (40%). We recorded bilateralism in just one corpse. The length of the arm measured from the acromion to the medial epicondyle of the humerus ranged between 9 and averaging 10.5 cm, averaging 10 cm. The length of the anastomotic branch ranged from 0.2 to 1.3 cm, with a mean of 0.60 cm. The nerve communication occurred in the upper third of the arm in seven limbs and the third medium in three arms (Figures 1 to ^6^).

**Figure 2:**
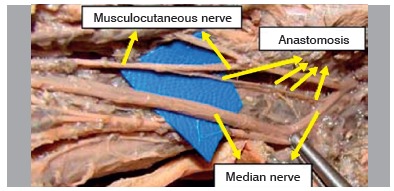
Specimen 12 - Right (presence of median-musculocutaneous anastomotic branch).

**Figure 3:**
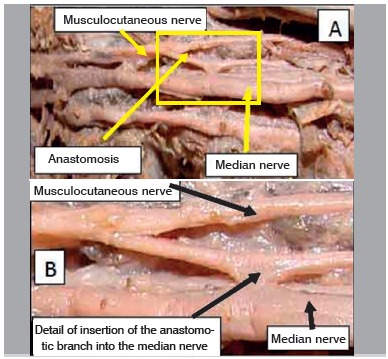
A. Specimen 16 - Left (presence of anastomotic branch); B. Detail of insertion of the anastomotic branch in the median nerve.

**Figure 4:**
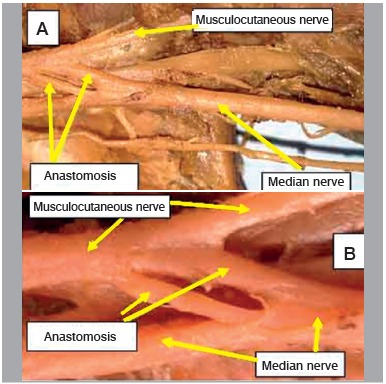
Specimen 26 - A. Left (presence of double anastomotic branch); B. detail of double anastomotic branch.

**Figure 5:**
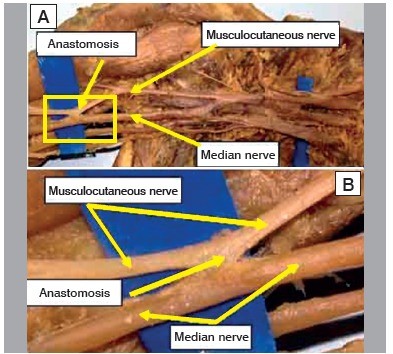
Specimen 16 - A. Left (presence of anastomotic branch in the middle third of the arm); B. Detail of the insertion of the anastomotic branch into the median nerve.

**Figure 6:**
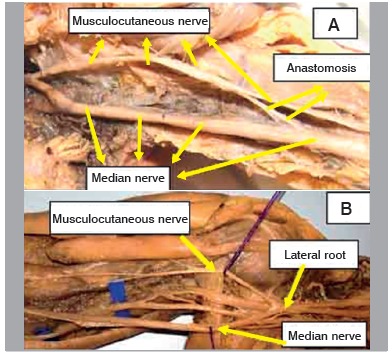
A. Specimen 29 - Left (presence of anastomotic branch in the middle third of the arm); B. Specimen 35 - Right (absence of anastomosis recorded in 30 limbs).

## DISCUSSION

Some authors^7^ ^-^ ^9^ reported MCN-MN communication incidence higher than 40% of cases, others^6^ ^,^ ^10^ ^-^ ^12^ less than 15%. We recorded a 25% incidence in limbs of dissected fetuses. Our results are close to those described by most as authors,^5^ ^,^ ^13^ ^-^ ^15^ since we recorded a nerve communication in 25% of limbs of dissected fetuses. We agree with Ballesteros et al.^5^ that multiple factors such as the sample size, the methodology used and the biological characteristics of the studied population may interfere with the variability of the results.

We recorded bilateral occurrence in just one specimen. The low incidence of bilateralism was also recorded by most authors.^7^ ^,^ ^11^ ^,^ ^16^ ^,^ ^17^ (Figure 6) The predominant incidence of only one communicating branch is registered by most studies in the literature. In only one limb we recorded the presence of more than two anastomotic branches. (Figure 4)

Most authors only mention that the communication branch goes from NMC to NM.^6^ ^,^ ^10^ ^,^ ^12^ ^,^ ^16^ ^-^ ^18^ However, NM communication NMC was observed for between 2.8% to 12.8%.^5^ ^,^ ^8^ We observed a MCN-MN communications in 90% of our dissections and in only one limb (10%) we observed MCN-MN communication.

Regarding the length of this anastomotic branch, Ballesteros et al.^5^ registered a mean of 57.8 mm and Loukas et al.^16^ 46 mm, while Elgseder and Goldman^14^ reported 18 mm. We worked with fetuses and recorded an average of 0.60 mm (the length of nerve communication). Uysal et al.^6^ also conducted studies evaluating the anatomical variations of the musculocutaneous nerve in fetuses, but they did not mention the length of nerve communications. We did not register any combination of MCN-MN communication with an additional brachial biceps head, as recorded by the authors.^5^ ^,^ ^7^ ^,^ ^8^ ^,^ ^19^

Knowledge on the existence of communication between MCN-MN is relevant for clinical practice. It allows assessment and appropriate management of motor disorders of the upper limbs caused by peripheral nerve lesions, and allows proper planning for the surgical approach. The musculocutaneous nerve, after passing under the pectoralis minor muscle goes into a narrow space, limited anteriorly by coracobrachialis muscle, and posteriorly by the upper third of the humerus. It can undergo dynamic compression at this location, causing paresthesia in the anterolateral surface of the forearm. This rare compression occurs in athletes and bodybuilders who have these muscles hypertrophied. The carriers of this compression can refer an inaccurate pain in the anterior face of the arm. If the compression become intense and lasts long, hypotrophy may occur at the anterior surface of the arm muscles (biceps and brachial). The presence of fibrous bands between the biceps and brachial muscles may compress the musculocutaneous nerve.^20^ If the compression occurs proximally to nerve communication, it may cause symptoms similar to carpal tunnel syndrome or even symptoms of less frequent compressive neuropathies such as the pronator teres syndrome or anterior interosseous nerve syndrome, depending on the nature of the fibers contained in nerve communication. Similarly, the musculocutaneous nerve damage proximal to nerve communication can cause muscle weakness of the flexor muscles of the forearm or the muscles of the thenar region with clinical signs that simulate a partial injury of the median nerve. It is essential to differentiate a partial or complete nerve injury. However, the correct identification of these variations is not always easy, because it requires accurate clinical examination and electroneuromyography examination.^21^ Changes recorded in electroneuromyography examination without clinical evidence is not sufficient to indicate a surgical procedure. Careful dissection during surgery can prevent the injury from an anastomotic branch. The detailed clinical examination aided by electromyographic methods can assist in the diagnosis and prevent unnecessary surgical procedures. Surgeons who perform neurotization procedures of the musculocutaneous nerve to restore elbow flexion should be aware of these anatomical variations.^22^

The lateral cutaneous nerve of the forearm is continuation of the musculocutaneous nerve. The forced flexion with the forearm in supination puts in tension the *lacertus fibrosus* and may compress the nerve, thus, triggering painful and paresthetic symptoms in the anterolateral aspect of forearm.^20^ The patient usually complains of burning pain in the anterolateral aspect of the elbow and forearm. The subject may assume an antalgic attitude with elbow flexion and forearm pronation, because the elbow extension and forearm supination tension the tendon of the biceps brachial muscle and the lateral cutaneous nerve of the forearm. Therefore, physical or occupational activity in pronosupination with the elbow in extension may trigger the symptoms. 

## CONCLUSION

The knowledge on nerve communication is of great significance, especially when considering the physical examination, diagnosis, prognosis and surgical treatment. If these variations are not valued, mistakes and consequences will be inevitable. The nerve communication between the musculocutaneous and median nerves can cause changes in clinical symptoms, especially in patients with carpal tunnel syndrome, since these variations may exacerbate or alleviate the symptoms causing motor and sensory changes different from the usual pattern.
